# Alcoholic Myopathy: Pathophysiologic Mechanisms and Clinical Implications

**DOI:** 10.35946/arcr.v38.2.05

**Published:** 2017

**Authors:** Liz Simon, Sarah E. Jolley, Patricia E. Molina

**Affiliations:** Liz Simon, M.V.Sc., Ph.D., is an Assistant Professor in the Department of Physiology, Alcohol and Drug Abuse Center of Excellence; Sarah E. Jolley, M.D., M.Sc., is an Assistant Professor in the Division of Critical Care Medicine; and Patricia E. Molina, M.D., Ph.D., is the Richard Ashman, Ph.D. Professor and Department Head of Physiology, and Director of the Comprehensive Alcohol-HIV/AIDS Research Center and Alcohol and Drug Abuse Center of Excellence, all at the Louisiana State University Health Sciences Center, New Orleans, Louisiana

**Keywords:** Alcohol consumption, alcohol use disorder, alcohol effects and consequences, alcoholic myopathy, skeletal muscle, skeletal muscle dysfunction, myopathy, muscle

## Abstract

Skeletal muscle dysfunction (i.e., myopathy) is common in patients with alcohol use disorder. However, few clinical studies have elucidated the significance, mechanisms, and therapeutic options of alcohol-related myopathy. Preclinical studies indicate that alcohol adversely affects both anabolic and catabolic pathways of muscle-mass maintenance and that an increased proinflammatory and oxidative milieu in the skeletal muscle is the primary contributing factor leading to alcohol-related skeletal muscle dysfunction. Decreased regenerative capacity of muscle progenitor cells is emerging as an additional mechanism that contributes to alcohol-induced loss in muscle mass and impairment in muscle growth. This review details the epidemiology of alcoholic myopathy, potential contributing pathophysiologic mechanisms, and emerging literature on novel therapeutic options.

Alcohol use disorder (AUD) affects approximately 15 to 20 million individuals in the United States (Center for Behavioral Health Statistics and Quality 2016), and excessive alcohol consumption is associated with $249 billion in economic costs (Sacks et al. 2015). Each year, alcohol consumption is linked to 2.3 million years of potential life lost, with over $150 billion attributable to effects of physical inactivity (Bouchery et al. 2011). Skeletal muscle dysfunction (i.e., myopathy) is common in patients with AUD, and alcoholic myopathy occurs in 40 to 60 percent of chronic alcoholics (Fernández-Solà et al. 2007; Urbano-Marquez and Fernández-Solà 2004). Although alcohol-related muscle disease is nearly 5 times more common than liver cirrhosis (which is present in 10 to 15 percent of people with AUD), data are lacking on its contribution to long-term health and disease in patients with AUD (Estruch et al. 1993). This review explores the epidemiology of alcohol-related myopathy, highlights the emerging literature on pathophysiologic factors associated with its development, and reviews novel targets for treatment.

## Epidemiology of Alcohol-Related Myopathy

Alcoholic myopathy is common among people with AUD and may manifest as an acute or chronic condition. Acute alcoholic myopathy is present in 0.5 to 2.0 percent of alcoholics, with an estimated overall prevalence of 20 cases per 100,000 people in the Western Hemisphere (Preedy et al. 2003). Chronic alcoholic myopathy is one of the most common types of myopathy, with an overall prevalence of 2,000 cases per 100,000 people. Based on these prevalence estimates, chronic alcohol-related myopathy is 10 times more common than the most common inherited myopathy (i.e., nemaline myopathy), which has a prevalence of 200 cases per 100,000 individuals, and 67 to 1,000 times more common than Duchenne’s muscular dystrophy with an estimated prevalence of 2 to 30 per 100,000 people (Preedy et al. 2003). However, it is difficult to ascertain the exact prevalence, because the spectrum of clinical disease in alcohol-related myopathy varies (Estruch et al. 1993). In a study of alcoholics without a known diagnosis of myopathy, up to 46 percent exhibited myopathic changes on muscle biopsies and presented with demonstrable reductions in strength compared with healthy control subjects (Urbano-Marquez et al. 1995). The role of this subclinical disease in the development of future clinically evident symptoms remains poorly understood.

The presence of liver cirrhosis also may influence the development of myopathy in people with AUD, because patients with cirrhosis secondary to chronic alcohol consumption commonly manifest muscle wasting. In a study of chronic alcoholic men, lean muscle mass was significantly lower in those with cirrhosis than in those without cirrhosis (Estruch et al. 1993). Lifetime ethanol consumption was an independent predictor of greater muscle loss among this population (Nicolas et al. 1993). Recent studies also suggest that the loss of muscle mass and strength associated with aging (i.e., sarcopenia) is more prevalent with advancing stages of cirrhosis and frequently occurs even in the absence of concomitant alcohol use (Hanai et al. 2016). The mechanisms involved in development and propagation of cirrhosis-related muscle disease are not completely understood and warrant further study; a further discussion is beyond the scope of this review.

## Clinical Manifestations

Clinically, acute alcoholic myopathy is characterized by weakness, pain, tenderness, and swelling of affected muscles. It often occurs after an alcohol binge characterized by consumption of 4 to 5 alcoholic drinks during a single episode, resulting in blood alcohol levels of 0.08 g/dL or above, and resolves within 1 to 2 weeks of abstinence from alcohol (Perkoff 1971). A common manifestation of acute alcoholic myopathy is a breakdown of muscle tissue and release of muscle-fiber content into the blood (i.e., rhabdomyolysis). It most severely affects muscles close to the body’s midline (i.e., proximal muscles), primarily the pelvic and shoulder girdles, in a focal and asymmetric fashion. Clinical evidence of this type of myopathy may be associated with laboratory evidence of muscle injury, accompanied by elevations in the enzyme creatinine kinase and the protein myoglobin that is found in heart and skeletal muscle. This so-called rhabdomyolytic variant of acute alcoholic myopathy, which in severe cases may precipitate acute renal failure, represents the most common nontraumatic cause of rhabdomyolysis in hospitalized patients (Urbano-Marquez and Fernández-Solà 2004).

Conversely, chronic alcoholic myopathy—the most frequent presentation of alcohol-related myopathy—presents with progressive proximal muscle weakness over weeks to months. Infrequently, patients experience pain, local muscle atrophy, muscle twitching, and/or muscle tightness (i.e., myotonia) (Urbano-Marquez and Fernández-Solà 2004). Chronic alcoholic myopathy is uncommon in patients under the age of 30 (Urbano-Marquez and Fernández-Solà 2004). Evidence of myopathy is associated with cumulative lifetime consumption of alcohol, with changes most evident with long-term, high-dose consumption (>10 kg of pure alcohol/kg of body weight) (Preedy et al. 2003; Urbano-Marquez and Fernández-Solà 2004). Thus, chronic alcohol-related myopathy occurs most commonly among people ages 40 to 60, with equal distribution between men and women. Chronic alcoholic myopathy has a higher incidence in patients with evidence of other alcohol-related organ dysfunction, occurring in 50 percent of patients with liver cirrhosis and 82 percent of those with alcohol-related heart muscle disease (i.e., cardiomyopathy) (Urbano-Marquez and Fernández-Solà 2004). Clinical series also indicate that patients with chronic alcoholic myopathy may be predisposed to presenting with episodes of acute alcoholic skeletal myopathy. Up to 30 to 46 percent of patients with a history of chronic alcohol abuse report episodic muscle pain (i.e., myalgia), weakness, and darkening of urine following an alcohol binge (Urbano-Marquez and Fernández-Solà 2004).

## Alcohol’s Effects on Mechanisms Controlling Muscle Mass and Function

### Altered Nutritional Status

Chronic heavy alcohol consumption can lead to protein calorie malnutrition, which frequently is related to the severity of alcoholic liver disease. One of the hallmarks of malnutrition resulting from chronic heavy alcohol consumption is a negative nitrogen balance, which can result from decreased nutrient intake and/or malabsorption of nutrients. As a result, micronutrient availability as well as circulating and tissue levels of growth factors are markedly altered following chronic alcohol consumption in animal models (Lang et al. 1998; Soszynski and Frohman 1992) and in humans (Röjdmark and Brismar 2001). Among the most frequently reported deficiencies in people with AUD are folate, thiamine, vitamin B6, zinc, and iron (Halsted 2004). Moreover, the incidence of vitamin D deficiency reportedly is greater in people with AUD than in healthy control subjects (Anty et al. 2015), and this has been suggested as a possible contributor to alcoholic myopathy (Wijnia et al. 2013). Although nutritional deficits likely are factors in alcoholic myopathy, a wealth of literature describes additional specific biochemical, metabolic, and epigenetic alterations that play important roles in the underlying pathophysiology of alcoholic myopathy. A brief summary of the most relevant mechanisms is provided below.

### Reduced Protein Synthesis

**Figure 1:**
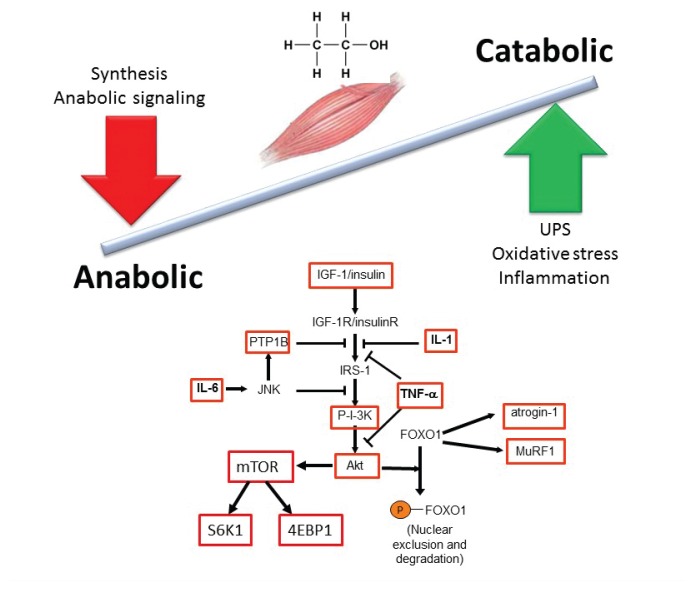
Principal effects of chronic alcohol abuse on anabolic and catabolic mechanisms that maintain skeletal muscle mass. Protein synthesis and breakdown are regulated by multiple factors, including anabolic hormones, nutrients, and myokines. Alcohol, depicted here by its chemical formulation, influences multiple aspects of both the anabolic and catabolic arms of the pathway. Numerous regulatory components of these pathways are altered by chronic alcohol exposure (see boxes). NOTE: 4EBP1 = eukaryotic initiation factor 4E binding protein; FOXO1 = Forkhead box protein O1; IGF-1 = insulin-like growth factor; IL-1 = interleukin 1; IL-6 = interleukin 6; IRS-1 = insulin receptor substrate 1; JNK = c-Jun N-terminal kinase; mTOR = mammalian target of rapamycin; MuRF1 = muscle RING-finger protein-1; P-I-3K = phosphatidylinositide 3-kinase; PTP1B = protein tyrosine phosphatase 1B; S6K1 = S6 kinase 1; TNF-α = tumor necrosis factor alpha; UPS = ubiquitin proteasome system.

A major mechanism contributing to decreased muscle mass in alcohol-related myopathy is the imbalance between protein synthesis (i.e., anabolic reactions) and protein breakdown (i.e., catabolic reactions). In particular, alcoholic myopathy is characterized by decreased protein synthesis (Steiner and Lang 2015) of both myofibrillar and sarcoplasmic proteins (Preedy and Peters 1988). Preclinical studies have identified several specific sites of alcohol-induced impairment in protein metabolism (figure 1). One of these is a protein called mammalian target of rapamycin (mTOR), which plays a central role in protein synthesis and is important for controlling skeletal muscle mass. It integrates signals from nutrients, growth factors, energy status, and stress and regulates cell size. mTOR is a subunit of two different protein complexes: mTOR complex 1 (mTORC1), which also contains a protein called Raptor and can be inhibited by a compound called rapamycin; and mTORC2, which also contains a protein called Rictor and is rapamycin insensitive. mTORC1 is the master regulator of protein synthesis. On stimulation, mTORC1 activates two parallel signaling pathways:

- It phosphorylates, and thereby activates, S6 kinase 1 (S6K1), whichleads to activation of the ribosomalprotein S6 that is required for protein synthesis (i.e., translation).
- It phosphorylates and activateseukaryotic initiation factor 4E (eIF4E)-binding protein (4EBP1). eIF4E is necessary for translation initiation; this process is inhibited by 4EBP1, which therefore acts as a translational repressor. Phosphorylation of 4EBP1 by mTORC1 releases this inhibition of eIF4E.

Chronic alcohol consumption has been shown to decrease activation of S6K1/S6 pathway and ribosomal protein S6 (rpS6) phosphorylation in skeletal muscle and in cultured muscle cells (i.e., myocytes) (Korzick et al. 2013). Alcohol also decreases the phosphorylation of 4EBP1 under both in vivo and in vitro conditions. This is associated with a redistribution of eIF4E from an active complex (i.e., eIF4E–eIF4G) to an inactive complex (i.e., eIF4E–4EBP1), thereby preventing mRNA translation. Finally, alcohol decreases the phosphorylation of mTOR itself (Lang et al. 2003) (figure 1). All of these effects contribute to decreased protein synthesis.

In addition to direct effects on the mTOR pathway, alcohol consumption significantly decreases levels of the insulin-like growth factor-1 (IGF-1) in both plasma and muscle, which is correlated with decreased muscle protein synthesis (Lang et al. 1998). Myocytes incubated with ethanol or its main metabolic products (i.e., acetaldehyde or acetate) show decreased IGF-1 and insulin-stimulated protein synthesis without significantly altering protein degradation (Steiner and Lang 2015).

**Figure 2:**
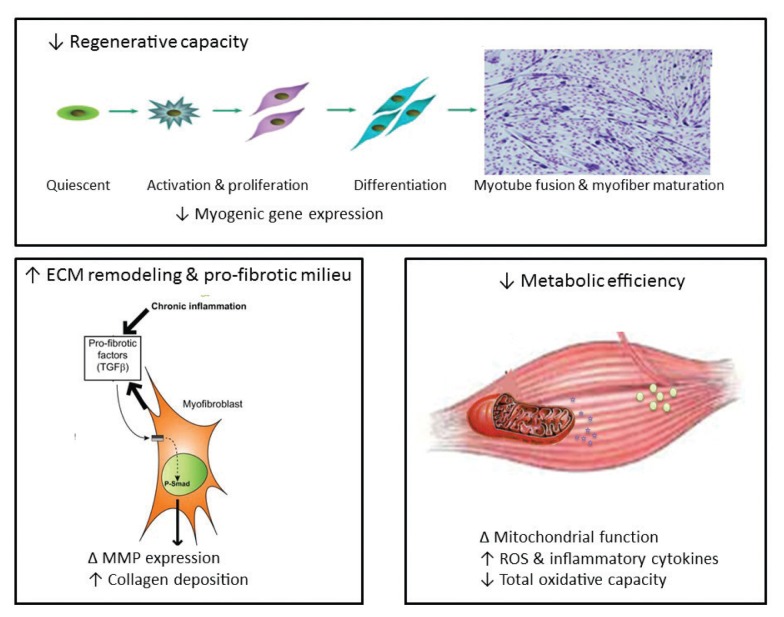
Mechanisms contributing to alcohol-induced loss of muscle mass and impairment in muscle growth. Decreased skeletal-muscle regenerative capacity is reflected as decreased myogenic gene expression, which prevents satellite-cell differentiation and myotube fusion and myofiber maturation. Chronic heavy alcohol consumption leads to skeletal-muscle inflammation, which favors expression of profibrotic factors such as transforming growth factor β(TGF-β), stimulating an increase in the expression and activation (phosphorylation) of transcription factors such as Smad (P-Smad). This in turn results in altered gene expression of matrix metalloproteinases (MMPs) and increased collagen deposition in the extracellular matrix (ECM) of skeletal muscle, which can prevent adequate satellite-cell activation, proliferation, and differentiation. Direct and indirect evidence indicates that alcoholic myopathy is associated with decreased mitochondrial function, enhanced reactive oxygen species (ROS) generation, and decreased total oxidative capacity, particularly in type 2 fibers. An increased proinflammatory and oxidative milieu in skeletal muscle likely is the underlying mechanism leading to the decreased regenerative capacity, development of a profibrotic milieu, and diminished metabolic efficiency.

An additional and alternative pathway controlling muscle mass involves the Smad family of proteins that act as transcription factors regulating the expression of several genes. These proteins are activated in response to transforming growth factor-beta (TGF-β), activin, and myostatin (figure 2). Smad signaling is required for myostatin-mediated inhibition of Akt/mTORC1 signaling, myotube atrophy, and TGF-β–induced fiber atrophy (Goodman and Hornberger 2014; McFarlane et al. 2006), all of which lead to a reduction of muscle mass. Alcohol exposure increases expression of insulin-like growth factor binding protein-1 (IGFBP-1) and myostatin, resulting in decreased skeletal muscle protein synthesis (Steiner and Lang 2015). Studies in macaques infected with the simian immunodeficiency virus (SIV) have demonstrated that chronic binge alcohol administration (CBA) increased myostatin mRNA levels, TGF-β levels, and fibrosis-promoting (i.e., profibrotic) gene expression in skeletal muscle at end-stage disease compared with sucrose-fed, SIV-infected macaques (Dodd et al. 2014; Molina et al. 2008). The increased expression of TGF-β and associated receptors and downstream signaling components could potentially decrease protein synthesis in addition to modulating extracellular matrix remodeling and promoting a profibrotic phenotype (Hong-Brown et al. 2015).

### Increased Protein Degradation

Protein degradation in skeletal muscle is directed primarily by two pathways, the ubiquitin proteasome pathway (UPP) and the autophagic–lysosomal system (Steiner and Lang 2015). Ubiquitin is a small protein found in almost all tissues of the organism. In the UPP, three enzymatic components are responsible for linking chains of ubiquitin to proteins destined for degradation. They include ubiquitin-activating enzymes (E1 enzymes), ubiquitin carrier or conjugating proteins (E2 proteins), and ubiquitin ligases (E3 ligases), which are also known as atrogenes. Two of these E3 ligases—atrogin-1 (also known as MAFbx) and MuRF1—are specific to the muscle. Proteins linked to the ubiquitin chains are recognized by a large protein complex called the 26S proteasome that is responsible for the degradation of intracellular proteins. This proteasome is composed of a 20S catalytic core, where actual protein degradation occurs, and two 19S polar caps that have regulatory functions.

Alcohol can interfere with normal functioning of the UPP in several ways, including both the proteasome itself and the ubiquitin binding to intracellular proteins. In the SIV-infected macaque model, CBA increased the expression of several proteins of the 19S caps, including S5A, Rpn6, and Rpn12 (LeCapitaine et al. 2011). Of these, Rpn6 is critical for assembly of the 19S cap and proper functioning of the 26S proteasome (Fernández-Solà et al. 2007). In rodents, both acute and chronic alcohol administration was associated with increased expression of the two E3 ligases atrogin and MuRF1 (Korzick et al. 2013; Vary et al. 2008). Elevated expression of MuRF1 and atrogin also has been found in a number of catabolic conditions affecting muscle and is thought to reflect increased UPP activity. Similarly, atrogin-1 expression was significantly increased in skeletal muscle at end-stage disease in CBA-administered, SIV-infected macaques (Molina et al. 2008). Moreover, the molecular changes in the UPP system protein expression were associated with increased proteosomal activity in skeletal muscle from end-stage, alcohol-treated, SIV-infected macaques (LeCapitaine et al. 2011). The findings are not universal, however, because studies in mice fed a liquid alcohol diet for about a month failed to show changes in the UPP pathway (Thapaliya et al. 2014).

The autophagic-lysosomal system is a protein degradation system activated by cellular stress that mediates breakdown of misfolded proteins. During this process, a double-membrane structure (i.e., phagophore) engulfs the proteins destined for degradation, as well as a portion of the cell’s cytoplasm, and then fuses to form an autophagosome. The mature auto-phagosome fuses with other vesicles (i.e., lysosomes) that contain enzymes which degrade the autophagosome contents (Shibutani and Yoshimori 2014); this is known as autophagy. There are contradicting data on the contribution or role of autophagy in alcohol-mediated muscle protein degradation. Thapaliya and colleagues (2014) have demonstrated increased expression of autophagy markers in the skeletal muscle of patients with alcoholic cirrhosis and in alcohol-fed mice. The study also demonstrated that in vitro treatment of C2C12 myotubes with 100 mmol/L alcohol (a concentration that exceeds physiological levels of alcohol) increased autophagic gene expression within 6 hours. Other investigators, however, did not observe a difference in autophagic gene or protein expression between chronic alcohol-fed mice and time-matched control animals (Steiner and Lang 2015; Steiner et al. 2015). Similarly, precursors of muscle cells (i.e., myoblasts) derived from CBA-treated macaques did not show changes in autophagic markers compared with myoblasts derived from sucrose-treated macaques (Simon et al. 2014). Thus, although decreased protein synthesis has been shown by several studies to play a role in alcohol-induced muscle loss, the contribution of catabolic pathways, particularly the UPP, also cannot be ignored.

## Mechanisms Implicated in Alcohol’s Effects on Factors Controlling Muscle Mass

Alcohol exposure seems to influence a variety of processes in the cells that may contribute to the altered protein synthesis and degradation levels described above. These include inflammatory reactions, oxidative stress, mitochondrial dysfunction, impaired muscle regeneration, as well as epigenetic and microRNA (miRNA)-related mechanisms (see figure 2).

### Inflammation

Acute alcohol intoxication reduces inflammation in response to infectious challenges; however, chronic alcohol consumption or administration promotes an inflammatory milieu, which may contribute to tissue injury (Molina et al. 2014). Alcohol-mediated increases in inflammation have been linked to oxidative stress as well as to organ damage or impaired function in muscle, brain, and cardiovascular and immune systems (Molina et al. 2014). Chronic inflammation also has been implicated as an underlying mechanism for loss of muscle mass. Proinflammatory cytokines such as tumor necrosis factor-α (TNF-α) and interleukin-6 (IL-6) may play a role in these processes (see figure 1). In chronic alcoholics, TNF-α was inversely related to lean mass, especially in the legs (Gonzalez-Reimers et al. 2011). Moreover, rodent studies demonstrated that chronic alcohol feeding led to a sustained increase in TNF-α and IL-6 mRNA as well as activation of a regulatory enzyme called JNK (Lang et al. 2014). Similarly, SIV-infected macaques that had received CBA developed a proinflammatory milieu in skeletal muscle with increased expression of TNF-α and IL-6 (Molina et al. 2006, 2008). Increased TNF-α expression increases protein degradation by the UPP in skeletal and cardiac muscle (Karlstad et al. 2000), supporting alcohol-induced chronic inflammation as an underlying mechanism that promotes skeletal muscle protein degradation and, consequently, loss of muscle mass.

### Oxidative Stress

Alcohol is primarily metabolized to acetaldehyde by alcohol dehydrogenase and cytochrome p450 2E1 (CYP2E1) in the liver. Alcohol oxidation by CYP2E1 is upregulated with chronic alcohol abuse and has been shown to produce a large amount of reactive oxygen species (ROS) (Cederbaum 2001). Alcohol metabolism, production of ROS, impaired antioxidant mechanisms, and changes in the cellular redox state all are well-known mediators of tissue injury in several organ systems (Fernández-Solà et al. 2007; Molina et al. 2014). Chronic alcohol-fed rats show reductions in several antioxidant systems, including total and free glutathione levels, glutathione reductase activity, glutathione peroxidase activity, and superoxide dismutase 2 activity (Otis et al. 2007). In addition, skeletal muscle exhibits increased protein carbonylation (Dekeyser et al. 2013) as well as elevated cholesterol hydroperoxide and malondialdehyde content (Fernández-Solà et al. 2007), all of which reflect oxidative injury. This increase in oxidative stress promotes protein degradation, including increased expression of the UPP system in myotubes (Gomes-Marcondes et al. 2002), and increases the expression of atrogin-1 and TGF-β1 (Otis et al. 2007). As discussed earlier, these factors work together to promote protein degradation and most likely impaired regeneration, resulting in muscle loss.

### Mitochondrial Dysfunction

Mitochondria are critical not only for providing the energy necessary for muscle contraction but also because they are involved in the regulation of redox homeostasis and integration of cell-death signaling (Marzetti et al. 2010). Most of the ROS formed in the cells are a byproduct of biochemical reactions (i.e., oxidative phosphorylation) in the mitochondria. At the same time, multiple defense mechanisms, including detoxifying enzymes and nonenzymatic antioxidant networks, are located in the mitochondria to cope with this physiological ROS production. However, excessive ROS generation, defective oxidant scavenging, or both have been implicated in mitochondrial dysfunction in sarcopenia and the pathogenesis of different myopathologies (Calvani et al. 2013; Lightfoot et al. 2015). Oxidative stress increases the incidence of mutations in the mitochondrial DNA (mtDNA), which is more susceptible to this damage than the DNA in the cell nucleus for several reasons. First, the mtDNA is located in close proximity to the site of ROS production, namely the electron transport chain (ETC). Second, the mtDNA’s repair system is less efficient compared with that of the nuclear DNA. Finally, mtDNA lacks noncoding sequences (i.e., introns) where mutations would have no effect on the final protein product; therefore, any damage to the mtDNA immediately affects the proteins encoded by that DNA. Oxidative-stress–induced mutations in the mtDNA, in turn, lead to defective ETC components, decreased ATP production, and more ROS generation (Calvani et al. 2013).

However, ROS damage not only the mtDNA but also lipids, including cardiolipin (DiMauro 2006), which is an integral part of the inner mitochondrial membrane and, among other functions, can trap protons generated in the ETC as well as can trigger apoptosis. Similarly, ROS may damage proteins that reduce ETC activity and promote apoptosis (Gonzalvez and Gottlieb 2007). In addition, oxidative stress activates transcription factors that mediate catabolic processes (McClung et al. 2010) and modify contractile elements, making them targets for protein breakdown (i.e., proteolysis) (Grune et al. 2003). All of these aspects of mitochondrial dysfunction can contribute to myopathies. However, mitochondrial myopathies are considered to be genetic defects that impair the synthesis, assembly, or maintenance of ETC components and involve primary mtDNA mutations as well as nuclear mutations that disrupt the replication of mtDNA, synthesis of ETC components, or mitochondrial protein synthesis (Sharp and Haller 2014).

The adverse effects of chronic alcohol abuse on mitochondrial function in skeletal muscle are unclear. In a study of chronic alcoholics, Cardellach and colleagues (1992) demonstrated that alcoholic myopathy is not associated with impaired mitochondrial energy supply. However, chronic alcohol ingestion leads to increased glycogen and lipid storage, enlarged and distorted mitochondria, and a dilated sarcoplasmic reticulum (Rubin et al. 1976), strongly suggesting mitochondrial dysfunction. Moreover, mitochondrial fusion is inhibited in skeletal muscle from alcohol-fed rats. This is indicated by a reduction in the outer mitochondrial membrane fusion protein mitofusin-1 (Eisner et al. 2014), a decline in mitochondrial integrity primarily resulting from dysfunction of the enzyme mitochondrial topoisomerase (Laurent et al. 2014), and a significant decrease in glycolytic enzymes and mitochondrial respiration rates (Trounce et al. 1990). Other studies found evidence that chronic alcoholics with clinical neurological manifestations exhibit lower levels of aerobic metabolic reactions (which require mitochondrial functions such as the ETC) but greater levels of anaerobic reactions (which do not require mitochondrial activity) (Haida et al. 1998), further indicating that chronic heavy alcohol consumption is associated with mitochondrial dysfunction. Additional mechanistic studies are warranted to determine whether this is caused by abnormal aerobic enzyme activity or other mitochondrial abnormalities. Regardless of the underlying mechanisms, mitochondrial abnormalities may ultimately result in impaired bioenergetics and disturbances in the process through which a nerve signal to the muscle leads to the muscle’s contraction (i.e., in excitation–contraction coupling of the muscle).

### Impaired Muscle Regeneration

Skeletal muscle injury triggers a well-defined healing or regenerative process involving inflammation, necrosis and degeneration of the affected tissue, activation of precursor cells (i.e., satellite cells), and subsequent regeneration of the muscle. Alcoholics are at an increased risk of several types of injuries, including those resulting from nerve damage to the limbs (i.e., peripheral neuropathies), falls caused by incoordination and imbalance, motor accidents, or muscle atrophy (Dekeyser et al. 2013). These injuries necessitate the activation of quiescent satellite cells, inducing them to proliferate and differentiate into myotubes to compensate for the enhanced skeletal muscle proteolysis and loss (Yin et al. 2013). Normally, during the initial inflammatory phase following skeletal muscle injury, two distinct subpopulations of immune cells called macrophages invade the injured muscle. The first population secretes inflammatory cytokines, such as TNF-α and IL-1. Subsequently, the proliferation and differentiation of satellite cells are facilitated by a second set of macrophages that secrete anti-inflammatory cytokines (Yin et al. 2013). Studies in SIV-infected male macaques have demonstrated that CBA results in accentuated upregulation of proinflammatory cytokine expression and depletion of antioxidant capacity in skeletal muscle, whereas administration of sucrose produces an overall skeletal muscle milieu that should trigger repair and regeneration capacity of satellite cells (LeCapitaine et al. 2011; Molina et al. 2008). More-over, myoblasts isolated from skeletal muscle samples obtained from CBA-treated macaques show a marked reduction in differentiation potential, which translates into decreased myotube formation, accompanied by decreased myogenic gene expression (Simon et al. 2014). These reports, as well as others in the literature, suggest that chronic alcohol administration or consumption may cause altered patterns of growth-factor and fibrotic gene expression in skeletal muscle (Dodd et al. 2014; Eisner et al. 2014), which may contribute to impaired regenerative capacity of muscle stem cells.

## Epigenetic and microRNA Alterations

In vivo and in vitro exposure to alcohol can modify gene expression through epigenetic mechanisms in several tissues, including the liver, brain, and immune system (Shukla and Lim 2013; Zakhari 2013). Emerging evidence also suggests that epigenetic modulation may mediate fetal alcohol spectrum disorders (Zakhari 2013). Epigenetic modulation involves chemical modifications of the DNA, such as methylation; histone modifications, such as methylation, acetylation and deacetylation, phosphorylation, addition of ubiquitin molecules (i.e., ubiquitinylation), addition of adenosine-diphosphate ribose (i.e., ADP-ribosylation), and addition of small ubiquitin-like molecules (i.e., sumoylation); as well as the actions of noncoding microRNAs (miRNAs). Unlike genetic alterations (i.e., mutations) or defects, epigenetic alterations do not alter the DNA sequence itself and can be reversed by therapy. Thus, elucidating alcohol-induced epigenetic changes opens new avenues for therapy of alcohol abuse and the resulting organ damage, such as the use of compounds that prevent histone deacetylation (i.e., histone deacetylase inhibitors), miRNA modulation, and similar approaches.

Alcohol exposure can induce epigenetic changes through several mechanisms. For example, alcohol metabolism in the liver, but also in other tissues (e.g., skeletal muscle), produces oxidative metabolites such as acetaldehyde, acetate, acetyl-CoA, and ROS, as well as nonoxidative products such as phosphatidylethanol and fatty acid ethyl ester (Curtis et al. 2013; Molina et al. 2014). Many of these products can induce tissue-specific epigenetic changes. For example, both in vivo and in vitro experiments have demonstrated that alcohol and its metabolites cause selective acetylation of histone H3 at a specific amino acid (i.e., the amino acid lysine at position 9 in the H3 molecule) (Kim and Shukla 2006).

Epigenetic mechanisms underlying alcohol-induced end-organ damage have been studied extensively in alcoholic liver disease. However, few studies to date have assessed alcohol-mediated epigenetic modulation specifically in the skeletal muscle. Ongoing studies are focusing on elucidating chronic alcohol-induced epigenetic and miRNA modifications that may contribute to impaired regeneration, metabolic dysregulation, and skeletal muscle wasting in people living with HIV/AIDS.

## Implications of Alcohol’s Effects on Muscle Mass and Function for Health and Disease

Human studies have demonstrated significant reductions in muscle mass associated with chronic alcohol consumption. Computerized tomography imaging of a region of the lower back (i.e., the L4 vertebrae) in a small cohort of chronic alcoholic subjects demonstrated a significantly reduced muscle area compared with healthy control subjects (Thapaliya et al. 2014). Similar results were found by Kvist and colleagues (1993) who noted significantly reduced femoral and gluteal muscle areas in chronic alcoholics, even though total lean body mass as determined from total potassium content did not differ significantly.

In contrast to the findings on muscle-mass changes in chronic alcoholics, analyses of the impact of alcohol consumption and abuse on exercise capacity have yielded conflicting results. Exposure to low doses of alcohol produced no effect on peak exercise capacity in healthy participants undergoing cycle ergometry or treadmill testing (Bond et al. 1984; Houmard et al. 1987). Similarly, reductions in peak strength measured by dynamometry were evident with moderate alcohol consumption, but not in individuals reporting lower alcohol consumption. However, consumption of higher doses of alcohol before exercise resulted in prolonged exercise times and failure to reach maximum oxygen consumption in healthy subjects (Lecoultre and Schutz 2009; McNaughton and Preece 1986). Thus, alcohol may have a dose-dependent effect on exercise-induced muscle changes (Barnes et al. 2010). Finally, studies of postexercise muscle function suggest that alcohol consumption may impair normal muscle remodeling after exercise-induced injury (Barnes et al. 2010).

Although an increasing number of studies are investigating the effects of alcohol use in healthy athletes, few studies have evaluated the long-term impact of chronic heavy alcohol consumption on muscle function. In survey studies, up to 15 percent of patients with AUD reported significant mobility impairments that occurred more frequently with greater alcohol disease severity and with the presence of alcohol-related comorbidities (Gossop et al. 2007; Gunther et al. 2007; Kim and Kim 2015). Studies of male patients enrolled in alcohol treatment programs also showed alterations in exercise capacity. For example, compared with age-adjusted control subjects, detoxified alcoholics demonstrated significant reductions in isokinetic torque, work, and power as well as isometric and isotonic muscle loading (Pendergast et al. 1990; York et al. 1999). Reductions in maximal isometric voluntary force measured by knee extension also were more pronounced in older recovering alcoholics (Pendergast et al. 1990; York et al. 1999).

## Therapeutic Options for Alcohol-Related Myopathy

**Figure 3:**
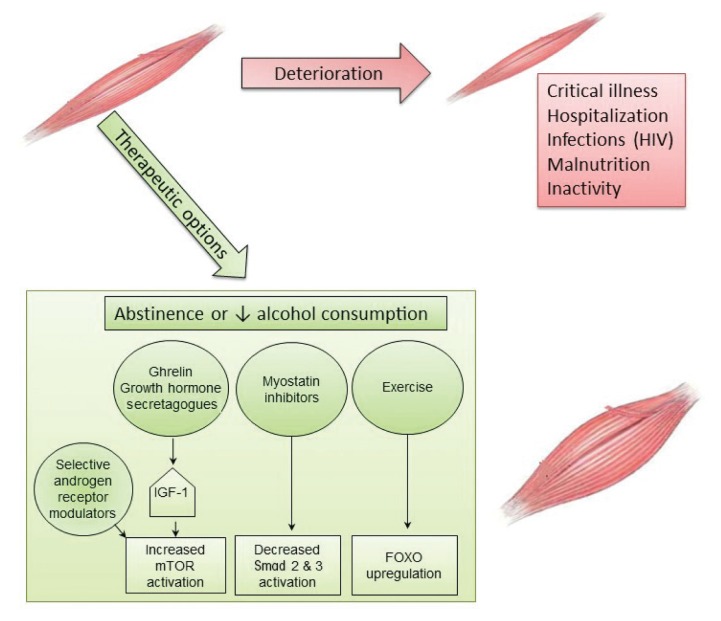
Aggravating conditions of, and therapeutic options for, alcoholic myopathy. Alcoholic myopathy may be further exacerbated by critical illness, prolonged hospitalization, chronic infection (e.g., HIV), malnutrition, and inactivity. Therapeutic options to achieve muscle mass accretion and restoration of skeletal muscle function include complete abstinence or at least decreased alcohol consumption, as well as aerobic exercise and/or resistance training. Other approaches currently being tested in myopathies of different etiologies also could prove effective for alcoholic myopathy. These include manipulation of the growth-hormone axis through administration of either insulin-like growth factor-1 (IGF-1), the principal mediator of growth-hormone action, or ghrelin, an upstream regulator of the growth hormone/mammalian target of rapamycin (mTOR) axis. Inhibition of myostatin, a negative regulator of muscle growth, may reduce Smad signaling, thereby preventing loss of muscle mass. Finally, exercise may lead to upregulation of Forkhead box protein O1 (FOXO1). Further studies are needed to determine the efficacy of these therapies for amelioration of alcoholic myopathy.

Currently, the only known effective treatment for alcoholic myopathy is complete abstinence from alcohol (see figure 3). Fortunately, up to 85 percent of patients with biopsy-proven alcoholic myopathy demonstrate objective functional improvement in muscle strength within the first year of alcohol-drinking cessation and complete normalization of strength by the fifth year of abstinence (Estruch et al. 1998; Fernández-Solà et al. 2000). Even for patients unable to completely abstain from alcohol, reduced cumulative alcohol consumption results in improvements in muscle strength over time (Estruch et al. 1998; Fernández-Solà et al. 2000). Acute alcoholic myopathy usually reverses within days or weeks of abstinence, whereas chronic myopathic changes usually resolve within 2 to 12 months (Peters et al. 1985). Moreover, nutritional optimization, including correction of vitamin and electrolyte deficiencies, is associated with greater improvement of muscle health (Urbano-Marquez and Fernández-Solà 2004).

Physiotherapy often is recommended in patients with acute or chronic alcoholic myopathy, although its benefit on myopathy resolution has not been studied rigorously. Use of physical-activity interventions, primarily aerobic exercise and/or resistance-training activities, has been shown to improve exercise capacity in AUD patients (Brown et al. 2014; Capodaglio et al. 2003). Although previous randomized controlled trials of exercise were not limited to patients with alcohol-related myopathy, they demonstrated significant improvements in maximal oxygen consumption and baseline heart rate in individuals with AUD subjected to standardized exercise interventions compared with control subjects (Brown et al. 2014; Capodaglio et al. 2003). Additional studies are needed to understand whether exercise interventions are particularly beneficial to patients with AUD and alcohol-related myopathy and to further elucidate the effects of exercise on alcohol-related muscle changes, particularly at the cellular and molecular levels.

Novel therapeutic agents increasingly are being explored for treatment of myopathic disease. Although to date many of these have not been studied in alcohol-related myopathy, they present exciting targets for potentially ameliorating the substantial burden of alcoholic myopathy. Agents targeting hormonal pathways, muscle-injury pathways, and vitamin deficiencies related to muscle disease are being actively investigated. Studies stimulating the growth hormone axis using injection of IGF-1 and IGF-1 binding complex in alcohol-fed rats have achieved restoration of muscle protein synthesis to basal control values (Lang et al. 2004). Other studies found that oral or intravenous administration of ghrelin, an upstream regulator of the growth hormone/mTOR axis, can help maintain lean muscle mass in patients with wasting (i.e., cachexia) related to cancer or chronic lung disease (Garcia et al. 2013; Miki et al. 2012). Treatment with agents that can inhibit myostatin function (i.e., myostatin antagonists) in heart failure and sarcopenia models resulted in a reduction in Smad signaling, preventing loss of muscle mass (Heineke et al. 2010; Murphy et al. 2011). Active trials of pharmacologic myostatin antagonists are in early-stage clinical investigations. Finally, there are conflicting data regarding the use of selective androgen receptor modulators to maintain muscle mass. Early (i.e., Phase 1 and 2) clinical studies of these agents in patients with cancer and age-related sarcopenia showed increased lean muscle mass; however, these findings could not be replicated in larger Phase 3 trials (Dalton et al. 2011; Dobs et al. 2013; Stewart Coats et al. 2011). Additional studies are needed to understand the role of selective androgen receptor modulators as single-drug or combination therapy for muscle wasting.

## Summary

Alcohol-related muscle disease is the most common clinical manifestation of AUD. Despite the high prevalence of disease, alcohol-related myopathy frequently is unrecognized. Most importantly, myopathy significantly contributes to long-term impairments in physical function and diminishes health-related quality of life for people with AUD. Therefore, research on the mechanisms underlying alcoholic myopathy and potential therapeutic approaches to ameliorate the disease, particularly in individuals with comorbid conditions, is extremely relevant. Researchers increasingly are recognizing the molecular pathways contributing to alcohol-induced muscle wasting, including reductions in mTOR-mediated protein synthesis and excessive protein degradation by activation of the UPP and autophagic–lysosomal system. In the settings of acute inflammation, oxidative stress, and/or mitochondrial dysfunction, exacerbation of these pathways is associated with accelerated muscle wasting. Clinical manifestations of muscle wasting include impairments in muscle dynamics (i.e., strength, power, and force) and loss of mechanical unloading, which also promote alcohol-related bone loss. Identification of these key pathways offers novel targets for therapeutics aimed at reducing the burden of alcohol-related muscle disease. Further studies are needed to understand the role of exercise and drug interventions, such as growth hormone regulators, myostatin antagonists, and androgen modulation, on alcohol-related muscle wasting.

## Glossary

Creatinine Kinase (CK): CK is the most widely used enzyme for diagnosing and monitoring muscle injury. It is located on the inner mitochondrial membrane of myofibrils and in muscle cytoplasm. CK catalyzes the transfer of a phosphate from phosphocreatinine to adenosine diphosphate (ADP), producing creatinine and adenosine triphosphate (ATP)
Cytokine: Small proteins released from cells that have a specific effect on the interaction between cells, on the communication between cells, or on the behavior of cells
Electron Transport Chain (ETC): Series of compounds that transfer electrons from electron donors to electron acceptors via reduction and oxidation. The ETC serves as the most productive pathway of cellular respiration in humans
Epigenetic: Modifications in gene function that do not involve changes in the DNA sequence
Extracellular Matrix: The collection of extracellular molecules (e.g., collagen) that are secreted by cells and provide structural and biochemical support to the surrounding cells
Histone: Any of a group of five small proteins occurring in the nucleus of eukaryotic cells that organize DNA strands into nucleosomes by forming molecular complexes around which DNA winds
microRNA (miRNA): A short, noncoding segment of RNA that suppresses gene expression by binding to complimentary segments of messenger RNA and interfering with protein formation during translation
Mitochondria: Cellular organelles found outside the nucleus that produce energy for the cell via cellular respiration
Myostatin: A skeletal-muscle protein that acts as a transforming growth factor to restrain muscle growth
Myotonia: Tonic spasm of one or more muscles
Necrosis: Localized death of living tissue
Redox State: Ratio of the concentration of oxidized species to the concentration of reduced species
Sarcoplasmic Reticulum: Specialized endoplasmic reticulum of cardiac and striated skeletal muscle that functions as a storage and release area for calcium
Satellite Cell: A stem cell that lies adjacent to a skeletal muscle fiber and plays a role in muscle growth, repair, and regeneration
